# Effects of Folic Acid on Secretases Involved in Aβ Deposition in APP/PS1 Mice

**DOI:** 10.3390/nu8090556

**Published:** 2016-09-09

**Authors:** Tian Tian, Dong Bai, Wen Li, Guo-Wei Huang, Huan Liu

**Affiliations:** Department of Nutrition and Food Science, School of Public Health, Tianjin Medical University, Tianjin 300070, China; tiantiantmu@126.com (T.T.); baidongtmu@126.com (D.B.); liwen828@163.com (W.L.); huangguowei@tmu.edu.cn (G.-W.H.)

**Keywords:** Alzheimer’s disease, folic acid, Aβ generation, secretase, microRNAs

## Abstract

Alzheimer’s disease (AD) is the most common type of dementia. Amyloid-β protein (Aβ) is identified as the core protein of neuritic plaques. Aβ is generated by the sequential cleavage of the amyloid precursor protein (APP) via the APP cleaving enzyme (α-secretase, or β-secretase) and γ-secretase. Previous studies indicated that folate deficiency elevated Aβ deposition in APP/PS1 mice, and this rise was prevented by folic acid. In the present study, we aimed to investigate whether folic acid could influence the generation of Aβ by regulating α-, β-, and γ-secretase. Herein, we demonstrated that folic acid reduced the deposition of Aβ42 in APP/PS1 mice brain by decreasing the mRNA and protein expressions of β-secretase [beta-site APP-cleaving enzyme 1 (BACE1)] and γ-secretase complex catalytic component—presenilin 1 (PS1)—in APP/PS1 mice brain. Meanwhile, folic acid increased the levels of ADAM9 and ADAM10, which are important α-secretases in ADAM (a disintegrin and metalloprotease) family. However, folic acid has no impact on the protein expression of nicastrin (Nct), another component of γ-secretase complex. Moreover, folic acid regulated the expression of miR-126-3p and miR-339-5p, which target ADAM9 and BACE1, respectively. Taken together, the effect of folic acid on Aβ deposition may relate to making APP metabolism through non-amyloidogenic pathway by decreasing β-secretase and increasing α-secretase. MicroRNA (miRNA) may involve in the regulation mechanism of folic acid on secretase expression.

## 1. Introduction

Alzheimer’s disease (AD) is the most common type of dementia. The most common initial symptom is gradual memory loss followed by impairment of other intellectual abilities. The amyloid-β protein (Aβ) was isolated from brains with amyloid deposition and it was subsequently identified as the core protein of neuritic plaques [1,2]. The major constituents of the amyloid plaques are well established and include the 4 kDa Aβ peptides, primarily Aβ42 and Aβ40. Reduction in Aβ42 in prodromal AD might slow the progression of the disease by affecting the rate of plaque formation [3]. 

Aβ is derived from amyloid-β precursor protein (APP) by sequential proteolytic cleavage. The enzymes in this process are essential for APP processing and Aβ production. Cleavage of APP by α-secretase precludes Aβ generation as the cleavage site is within the Aβ domain, and releases a large soluble ectodomain of APP called sAPPα as well as a carboxyl terminal fragments (CTFs) of APP called αCTF. After α-cleavage, αCTF will be further cleaved by γ-secretase to puduce p3 peptides and APP intracellular domain (AICD). The pathway is known as “non-amyloidogenic”, since α-secretase processing of APP prevents the formation of intact Aβ peptide. The best-studied α-secretases were members of the ADAM (a disintegrin and metalloprotease) family, in particular ADAM9 and ADAM10 [4]. 

An alternative route that is particularly present in neurons and brain is known as the “amyloidogenic pathway” which involves cleavage of APP by β-secretase[beta-site APP-cleaving enzyme 1 (BACE1)]. This cleavage generates a truncated APP secreted form (sAPPβ) and a membrane-anchored carboxyterminal fragment of 99 amino acids (C99) that contains the entire Aβ region. C99 is subsequently cleaved by γ-secretase to generate the C-terminus of the Aβ peptide and AICD [5]. 

γ-secretase is a large complex composed of the four polypeptides: presenilin (PS1 or PS2), nicastrin (Nct), presenilin enhancer 2 (Pen2), and anterior pharynxdefective 1 (Aph1). γ-secretase is responsible for the processing of more than 70 transmembrane proteins involved in normal cellular processes, including regulation of cell fate, cell adhesion, migration, neurite outgrowth, synaptogenesis, calcium homeostasis, transport of membrane proteins, and cell signaling [6].

Briefly, amyloidogenic processing involves β- and γ-secretases which induce Aβ production. Nonamyloidogenic processing mainly occurs at the cell surface, where α-secretase is particularly enriched. 

MicroRNAs (miRNAs) are 20- to 22-nucleotide RNAs that regulate gene expression at the posttranscriptional level by binding with partial complementarity to the 3′ untranslated region (UTR) of target messenger RNAs (mRNAs), thereby leading to translational inhibition or degradation [7]. miRNAs are essential for neuronal development, function, and survival. Accumulating evidence in AD studies suggests that the alterations in the miRNA network could contribute to the risk for AD [8]. Some studies have revealed that those secretases mentioned above can be regulated by miRNAs. For examples, ADAM9 is the target of miR-126 [9,10]. ADAM10 is the target of miR-665 [11]. miR-24, miR-186, and miR-455 are regulators of NCT [12]. BACE1 is the target of miR-186 and miR-339 [13,14].

A few randomized clinical trials have demonstrated folate, administered in isolation or in combination with other B vitamins, may improve the memory or social function in older individuals with neuropsychiatric alterations [15,16,17]. In previous studies, we have found folate deficiency elevated hippocampal APP, PS1 and total Aβ protein levels in APP/PS1 mice, and these rises were prevented by folic acid [18]. Furthermore, we have discovered folic acid deficiency can decrease several amyloid-associated miRNAs expression which may target APP and BACE1 [19]. PS1 and BACE1 are both important secretases involved in Aβ production, so we hypothesized that folic acid may influence the expression of multiple secretases related to APP processing and Aβ accumulation. Some other researches support our hypothesis. For example, deprivation of folate and vitamin E, coupled with dietary iron could increase PS1 expression, γ-secretase and Aβ generation in ApoE-/- mice [20]. A combined folate, B12 and B6 dietary deficiency, would induce amyloid-beta overproduction and PS1/BACE up-regulation in TgCRND8 and 129Sv mice [21]. However, whether folic acid supplementation regulates secretases is still unknown. Therefore, in the present study, we further investigate the changes of Aβ42 and Aβ40, α-, β-, and γ-secretase, as well as miRNAs, may target certain secretases in APP/PS1 mice brain tissue under folate deficiency and two levels of folic acid administration.

## 2. Materials and Methods

### 2.1. Animals and Diet

The Tianjin Medical University Animal Ethics Committee approved the experimental protocols in this study (Study number: TMUaMEC 2012016). Male mice with APPswe/PS1ΔE9 mutations (APP/PS1), backcrossed to C57Bl6/J, were obtained from the Chinese Academy of Medical Sciences Institute of Laboratory.

After genotyping, the AD transgenic mice were maintained on the control diet until the age of seven months and then were assigned in equal numbers to four groups: (1) folate-deficient diet plus daily gavage with water (deficiency); (2) control diet (normal folic acid content) plus daily gavage with water (control); (3) control diet plus daily gavage with 120 μg/kg folic acid (120 μg/kg); and (4) control diet plus daily gavage with 600 μg/kg folic acid (600 μg/kg).

The folate-deficient diet (containing folic acid 0.2 mg/kg diet) and the control diet (folic acid 2.1 mg/kg diet) were purchased from TestDiet (St. Louis, MO, USA). Thus, the average folic acid intake level for the four groups are 1 μg/day, 10.5 μg/day, 15.3 μg/day, and 34.5 μg/day, respectively.

All mice received food and drinking water ad libitum. Diets were treated for eight weeks. At the conclusion of the experiment, the mice were anesthetized by intraperitoneal injection of 7% chloral hydrate (5 mL/kg) and perfused transcardially with phosphate buffered saline (PBS).

Brains were removed, bisected in the sagittal plane, and stored at −80 °C. Left brain tissue was used for immunohistochemistry staining and right brain tissue was used for other assays, as described below.

### 2.2. Serum Folate

Serum folate levels were determined by using a competitive protein-binding assay with chemiluminescent detection in an automated chemiluminescence system (Immulite 1000; Siemens, Berlin, Germany) according to the manufacturer’s instructions at the end of the eighth week. The automated chemiluminescence system would detect all types of folate, including folic acid, dihydrofolate, and tetrahydrofolate.

### 2.3. Immunohistochemistry

The brains were removed and post-fixed with 4% paraformaldehyde in 0.1 M phosphate buffer (pH 7.4) at 4 °C overnight. The brains were coronally-cut into 4 μm-thick sections with a vibratome. Free-floating sections were incubated with 4% bovine serum albumin in PBS for 1 h, then reacted with monoclonal anti-β-amyloid protein antibody (Bam10, 1:3000, Sigma Aldrich, A5213; St. Louis, MO, USA) at 4 °C overnight. The sections were washed with PBS and reacted with biotinylated secondary antibodies diluted 1:200 in PBS and visualized using ABC Elite kit (Vector Laboratories, Burlingame, CA, USA). The images were carried out using a microscope (Olympus, Tokyo, Japan) and the integrated optical densities (IOD) of the hippocampi were determined with Image-Pro Plus 6.0 (Media Cybernetics, Rockville, MD, USA, 2003). 

### 2.4. ELISA for Aβ Quantification and Secretases Activity

Levels of Aβ40 and Aβ42 in mice brain were determined with ELISA assay by using mouse β-Amyloid 40 and 42 ELISA kit (Invitrogen, Carlsbad, CA, USA). α- and β-secretase activities were measured by relevant kits according to the manufacturer’s instructions (R and D Systems). Optical density (OD) was read at 450 nm within 30 min on a microplate spectrophotometer (Denley Dragon Wellscan MK3) (Thermo Fisher Scientific, Vantaa, Finland). Concentrations were calculated according to the standard curve. The percentage of concentration in the control group was shown.

### 2.5. Western Blot Analysis

Protein expression of ADAM9, ADAM10, BACE1, PS1, and NCT in mice brain was assessed by Western blot analysis. Total protein was extracted from homogenized brains with extraction buffer. Protein concentrations in the supernatants were determined by BCA protein assay kit (Thermo Scientific, Vantaa, Finland), using bovine serum albumin as a standard. Equal amounts of protein were loaded in each well for sodium dodecyl sulfate 12% polyacrylamide gel electrophoresis and then the separated proteins were transferred to nitrocellulose membranes. The membranes were blocked with 5% non-fat milk and incubated with primary antibodies (anti-ADAM9, 1:1000, CST; anti-ADAM10, Abcam; anti-BACE1, 1:1000, CST; anti-PS1, 1:1000, Abcam; anti-NCT, 1:1000, CST) overnight at 4 °C. Membranes were rinsed three times with Tris-buffered saline with Tween 20 (TBST) before being incubated with horseradish peroxidase-conjugated secondary antibody (1:10,000 in TBST) for 2 h and detected by chemiluminescence. Quantitation of proteins was done by densitometric analysis using NIH Image software (version 1.61, National Institutes of Health, Bethesda, MD, USA, 1997). The intensity of each protein band was normalized to the respective actin band (anti-β-actin, 1:5000, Abcam).

### 2.6. Real-Time PCR for mRNA and miRNA Expression

For mRNA detection, total RNA was extracted using TRIzol. First-strand cDNA was synthesized from 2 μg total RNA by using MMLV reverse transcriptase. The 20 μL reaction volume was incubated for 50 min at 42 °C, 5 min at 90 °C, 5 min at 5 °C and was then stored at −20 °C. Real-time PCR was performed using the LightCycler 490 SYBR Green I Master Kit (Roche, Mannheim, Germany). The 20 μL PCR mixture included 10 μL of PCR Master mix, 5 μL of cDNA, 1 μL of forward primer, 1 μL of reverse primer, and 3 μL of water. The reaction mixtures were incubated at 95 °C for 5 min, which was followed by 45 amplification cycles (denaturation, 95 °C for 10 s; annealing, 56 °C for ADAM9 and ADAM10, 59 °C for BACE1 and 63 °C for PS1 for 10 s; extension, 72 °C for 10 s). Primers were specific for ADAM9 (forward, GCTGTCTTGCCACAGACCCGGTATGTGGAG; reverse, TGGAATATTAAGAAGGCAGTTTCCTCCTTT), ADAM10 (forward, AGTAGTAATCCAAAGTTGCCG; reverse, GTGTCCCATTTGATAACTCTCTC), BACE1 (forward, AATGTGCCTGTGGTTGTAGTC; reverse, TTGAAGAAGCAGAGAGAGACAG), and PS1 (forward, GCCCCAGAGTAACTCAAGACA; reverse, CCGGGTATAGAAGCTGACTGA). The assay was performed using the Roche LightCycler 480 sequence detector (Roche). The expression of each gene was normalized to β-actin (forward, AATGTGTCCGTCGTGGATCT; reverse, GGTCCTCAGTGTAGCCCAAG) in order to calculate relative levels of transcripts.

For miRNA determination, total RNA was reverse-transcribed to cDNA using RNase Inhibitor (Epicentre, Madison, WI, USA), dNTP (HyTest Ltd., Turku, Finland), RT buffer, and RT primers (Invitrogen). The mixture was incubated at 16 °C for 30 min, 42 °C for 40 min, and 85 °C for 5 min to generate a library of miRNA cDNAs. U6 is used as an internal control for normalization. Real-time PCR was subsequently performed using an ABI PRISM7900 system (Applied Biosystems, Foster City, CA, USA) according to a standardized protocol. The reactions were incubated at 95 °C for 10 min, followed by 40 cycles at an interval of 10 s at 95 °C and an interval of 60 s at 60 °C. Data were analyzed by 2^−ΔΔCT^.

### 2.7. Statistical Analysis

Results were expressed as mean ± S.D. and analyzed using the SPSS 13.0 software package (SPSS Inc., Chicago, IL, USA, 2004). Data were analyzed by a one-way ANOVA followed by a Student–Newman–Keuls test. *p* values < 0.05 were considered to be statistically significant.

## 3. Results

### 3.1. Folic Acid Reduces Total Aβ Deposition and Aβ 42 Protein Level, Increases Serum Folate Level

The aim of this study was to determine the effects of folate deficiency and supplementation on AD-like pathology in APP/PS1 mice when daily treatment begun at seven months of age, when visible Aβ deposition may begin to be detected in this mouse model.

**Figure 1:**
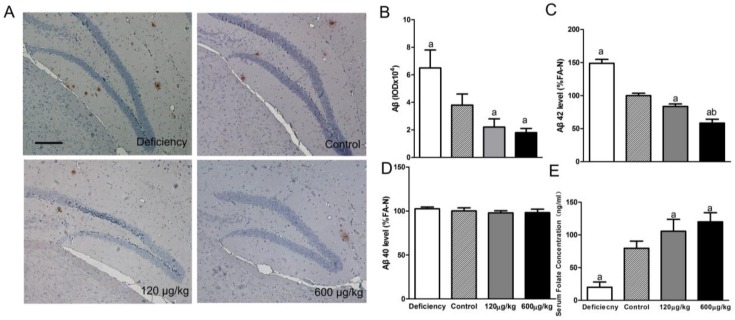
Folate reduced hippocampal amyloid plaque loads in APP/PS1 (amyloid precursor protein)/(presenilin 1) mice. With bam-10 immunohistochemical staining following the administration, compared with the control group, the deposition of Aβ was significantly decreased in both 120 μg/kg and 600 μg/kg groups (**A**,**B**); scale bar = 100 μm. Enzyme-linked immuno sorbent assay (ELISA) was used to measure Aβ levels in brain tissues. Compared with the control group, folic acid administration decreased Aβ42 deposition, but not Aβ40 (**C**,**D**). The Aβ42 level was further decreased in the 600 μg/kg group compared to the 120 μg/kg group. After eight weeks, the folate deficiency diet reduced serum folate. Folic acid administration increased serum folate compared with the control group (**E**). The data were expressed as means ± SD values, *n* = 6 animals/group. a: *p* < 0.05 versus the Control group; b: *p* < 0.05 versus the 120 μg/kg group.

Immunohistochemical analysis showed that the accumulated Aβ levels in brain tissue were higher in the deficiency group and lower in two folic acid supplementation groups than that in the control group (F[3,20] = 10.16, *p* < 0.05; Figure 1A,B).

ELISA test results demonstrated that, compared to the control group, the levels of Aβ42 deposits in the brain in the deficiency group was significantly higher, the Aβ42 deposits in both two folic acid administration group were lower (F[3,20] = 60.21, *p* < 0.05; Figure 1C). At the same time, we did not find a difference in Aβ40 accumulation in all AD groups (F[3,20] = 1.031, *p* > 0.05; Figure 1D).

Serum folate was detected at the end of eighth week. Compared to the control group, a folate deficient diet caused lower serum folate, and folic acid supplementation increased serum folate significantly (F[3,20] = 66.01, *p* < 0.05; Figure 1E). However, no significant difference in serum folate was shown between the 600 μg/kg and 120 μg/kg groups.

### 3.2. Folic Acid Increases ADAM9/ADAM10 Expression and α-Secretase Activity

**Figure 2:**
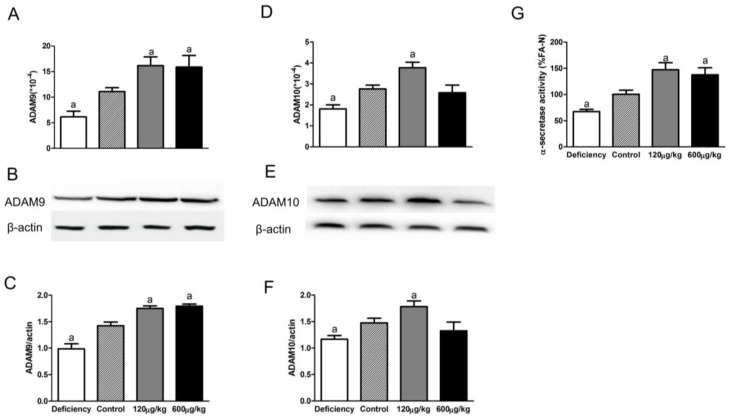
Folate stimulated ADAM9 and ADAM10 expression in APP/PS1 mice. The mRNA/protein levels of ADAM9 and ADAM10 in the brains of APP/PS1 mice were detected by qRT-PCR/Western blot analysis. Representative immunoblotting images of ADAM9 and ADAM10 are shown. Quantitative analysis revealed that the 120 μg/kg folic acid treatment led to up-regulation of ADAM9 (**A**–**C**) and ADAM10 (**D**–**F**). Folate deficiency led to down-regulation of ADAM9 and ADAM10. The 600 μg/kg folic acid treatment only up-regulated ADAM9, but did not change ADAM10 level; total α-secretase activity was reduced in the deficiency group and was increased in both 120 μg/kg and 600 μg/kg groups (**G**). The data were expressed as means ± SD values, *n* = 6 animals/group. a: *p *< 0.05 versus the control group.

As shown in Figure 2A–C, there was significant difference between four groups for ADAM9 expression (F[3,20] = 9.098, *p* < 0.05 for mRNA, F[3,20] = 31.4, *p* < 0.05 for protein). Compared to the control group the expression of ADAM9 mRNA and protein significantly decreased in the deficiency group, and increased in both the 120 μg/kg and 600 μg/kg groups. There was no difference between those two administration groups.

Similarly, folate deficiency and supplementation influenced the expression of ADAM10 (F[3,20] = 10.72, *p* < 0.05 for mRNA, F[3,20] = 9.348, *p* < 0.05 for protein; Figure 2D–F). The ADAM10 mRNA and protein expression decreased in the deficiency group and increased in the 120 μg/kg group compared to the control group. However, the expression of ADAM10 in 600 μg/kg group was not up-regulated.

Total α-secretase activity was further determined by ELISA. Results showed folic acid deficiency decreased α-secretase activity and folic acid supplementation increased α-secretase activity compared to the control group (F[3,20] = 12.11, *p* < 0.05; Figure 2G).

### 3.3. Folic Acid Inhibited BACE1 Expression and Activity

**Figure 3:**
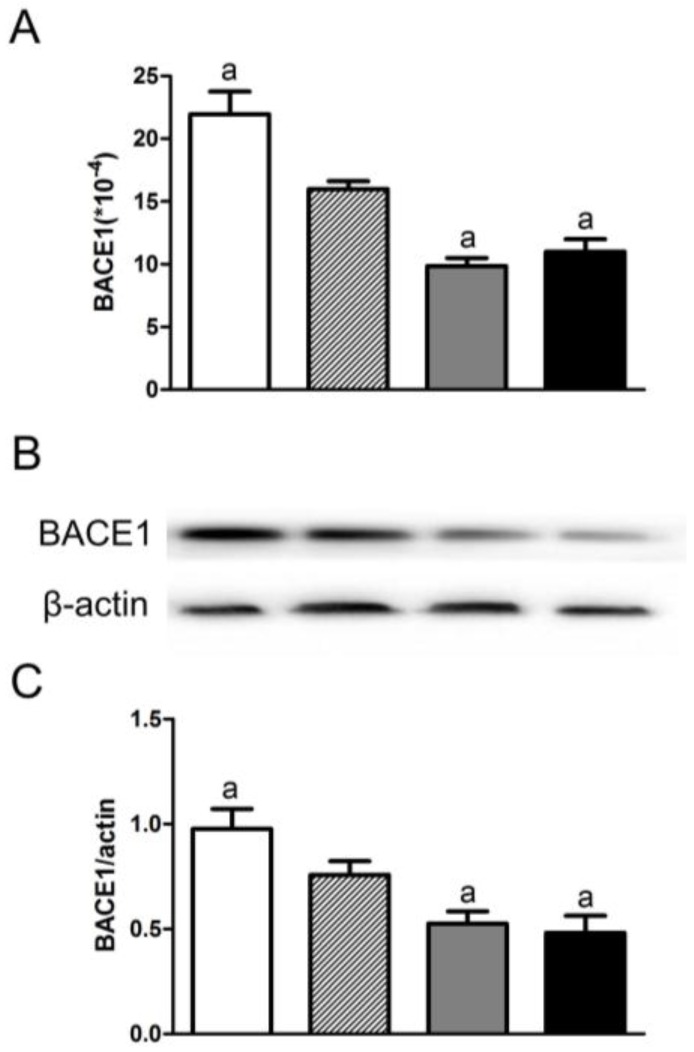

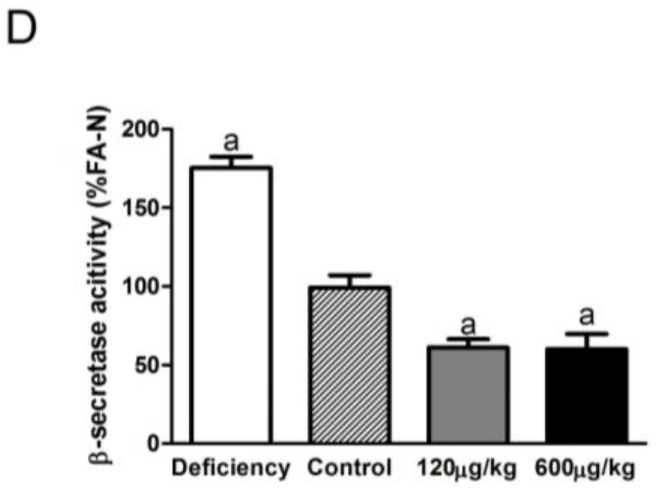
Folate inhibited BACE1 expression in APP/PS1 mice. BACE1 mRNAs and proteins in the brains of APP/PS1 mice were evaluated by qRT-PCR and Western blot analysis. The β-secretase activity was determined by ELISA. Representative bands and quantitative analysis revealed that folic acid treatment led to down-regulation of mRNA (**A**) and protein (**B**,**C**) expression of BACE1 and its activity (**D**). Folate deficiency led to up-regulation of BACE1 expression and activity. The data were expressed as means ± SD values, *n* = 6 animals/group. a: *p* < 0.05 versus the control group.

Compared to the control group, the mRNA/protein expression level and the activity of BACE1 increased significantly in the deficiency group and decreased in both folic acid supplementation groups (F[3,20] = 24.06, *p* < 0.05 for mRNA, F[3,20] = 8.917, *p* < 0.05 for protein, F[3,20] = 50.62, *p* < 0.05 for activity; Figure 3). There was no difference for BACE1 expression levels and its activity between the 120 μg/kg and 600 μg/kg group.

### 3.4. Folic Acid Influenced PS1 Expression but Not NCT Level

**Figure 4:**
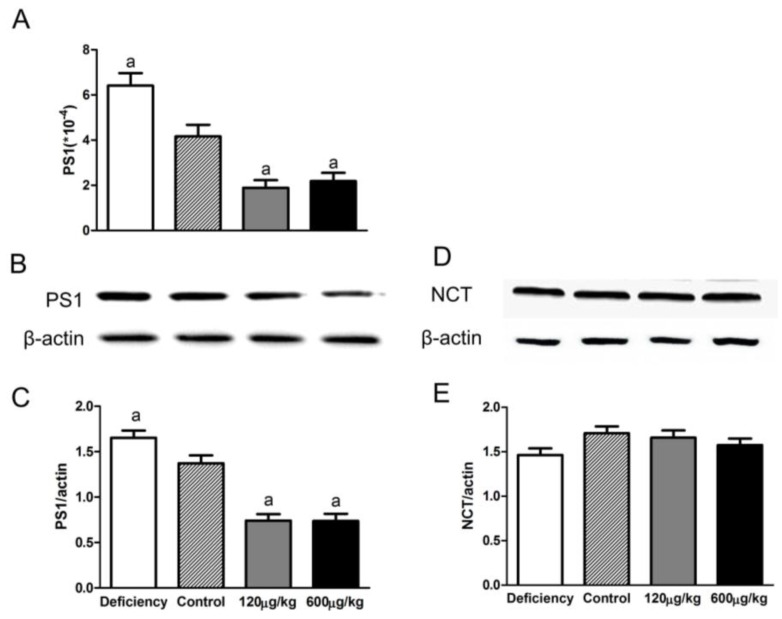
Folate inhibited PS1 expression, but not NCT (nicastrin), in APP/PS1 mice. PS1 mRNAs and proteins in the brains of APP/PS1 mice were determined by qRT-PCR and Western blot analysis. Folic acid treatment inhibited PS1 mRNA and protein expression. Folate deficiency led to up-regulation of PS1. No difference in PS1 expression was showed between 120 μg/kg and 600 μg/kg groups (**A**–**C**); However, Western blot analysis revealed that folic acid did not change NCT protein levels (**D**,**E**). The data were expressed as means ± SD values, *n* = 6 animals/group. a: *p* < 0.05 versus the Control group.

Compared to the control group, PS1 mRNA and protein levels increased in the deficiency group and decreased in the two supplementation groups (F[3,20] = 21.49, *p* < 0.05 for mRNA, F[3,20] = 34.23, *p* < 0.05 for protein; Figure 4A–C). No significant difference was found between the 120 μg/kg and 600 μg/kg groups. No significant difference showed in all groups for NCT protein (F[3,20] = 1.940, *p* > 0.05; Figure 4D,E).

### 3.5. Folic Acid Regulated miRNAs Related to ADAM9 and BACE1

**Figure 5:**
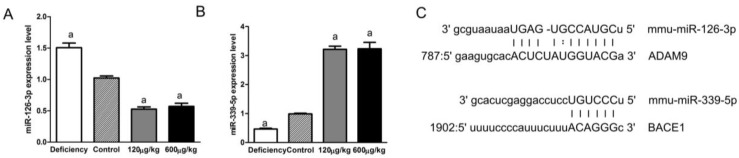
Folate regulated miR-126-3p and miR-339-5p expression in APP/PS1 mice brains. Two miRNA expression levels in the brains of APP/PS1 mice were confirmed by qRT-PCR. (**A**) miR-126-3p was expressed at lower level in both 120 μg/kg and 600 μg/kg groups, it was expressed at higher levels in the deficiency group compared to the control group; (**B**) miR-339-5p was expressed at higher level in both the 120 μg/kg and 600 μg/kg groups, it was expressed at lower level in the deficiency group compared to the control group; and (**C**) predicted miR-126-3p and miR-339-5p target sites in the 3′-UTR of ADAM9 or BACE1. Schematic representation of base pair matching between miRNAs and the 3-UTR of ADAM 9 and BACE1. The seed region of the miRNAs is indicated. The data were expressed as means ± SD values, *n* = 6 animals/group. a: *p* < 0.05 versus the control group.

miR-126-3p has already been identified as a miRNA which targets ADAM9 and can be regulated by DNA methyltransferases (DNMTs). miR-339-5p possibly target the seed same region on 3′-UTR of BACE1. In our study, miR-126-3p was up-regulated in the deficiency group and was down-regulated in both the 120 μg/kg and 600 μg/kg groups compared to the control group (F[3,20] = 84.41, *p* < 0.05; Figure 5A). On the contrary, miR-339-5p was down-regulated in the deficiency group and was up-regulated in both 120 μg/kg and 600 μg/kg groups compared to the control group (F[3,20] = 138.1, *p* < 0.05; Figure 5B). No significant difference was observed between the 120 μg/kg and 600 μg/kg groups.

## 4. Discussion

Our previous studies have observed the effects of folic acid on elderly people with mild cognitive impairment, animal, and cell models of AD. Those results showed a beneficial effect from folic acid supplementation on cognitive functioning in later life [22]. It was also demonstrated that folic acid may modify Aβ accumulation in vivo [18,20] and in vitro [23,24,25]. In the present study, we investigated whether α-, β-, and γ-secretase are all involved the inhibition effect of folic acid on Aβ accumulation.

We have demonstrated folic acid deficiency increased Aβ42 but not Aβ40 in APP/PS1 mice brain [19]. Moreover, the present study revealed that folic acid supplementation still decreased Aβ42 compared to Aβ40. Meanwhile, serum folate showed a dose-dependent trend in those four groups. Aβ42 is more amyloidogenic than Aβ40, and is deposited earlier than Aβ40 in the brain parenchyma in AD patients [26]. Aβ42 is the major isoform in the amyloid plaque in the brain of AD [27]. Since Aβ42 is relatively insoluble in the interstitial fluid and is prone to parenchymal deposition, whereas Aβ40 is more soluble and less prone to parenchymal deposition [28] might constitute the possible reason by which folic acid decreased Aβ42, and not Aβ40, in AD mice brains.

Two major pathways are involved in APP metabolism, one non-amyloidogenic and one amyloidogenic [29]. In the non-amyloidogenic pathway, APP is cleaved by α-secretase and releases sAPPα and CTF-α. γ-secretase cleaves CTF-α to produce p3 and AICD [30]. Our present work showed that folic acid increased α-secretase activity which itself was achieved by up-regulating the expression of the ADAM9 and ADAM10 mRNA and protein.

In the amyloidogenic pathway, APP is first hydrolyzed by BACE1 and generates sAPPβ and CTF-β. γ-secretase further cleaves CTF-β to release AICD and Aβ, which aggregates to form amyloid plaques [31]. BACE inhibition has been proposed to decrease the amount of APP processed into Aβ, and shunt APP to the α-secretase pathway. BACE1 is considered as an attractive drug target for reducing cerebral levels of Aβ for the treatment or prevention of AD [32,33]. Our study indicated that folic acid significantly decreased BACE1 mRNA and protein expression, suggesting that folic acid may be a BACE1 inhibitor which results in higher metabolism of APP through non-amyloidogenic pathway and reduction of Aβ generation. 

Reports document that γ-secretase has been considered as a multi-subunit aspartyl protease that cleaves APP and has proved to be a highly tractable target for AD drug treatment [34]. Our study showed that folic acid treatment could modify PS1, which is one of the subunits of γ-secretase. However, NCT expression was not regulated by folic acid in our results. γ-secretase activity is dependent on the interaction with all four essential γ-secretase components. Our study did not find enough evidence to suggest folic acid influencing γ-secretase activity. 

Several studies have revealed certain APP secretases can be modified by miRNAs [35,36]. Since folic acid regulated ADAM9 significantly in the present study, and ADAM9 has been demonstrated functioning as a direct target of miR-126 [37], we determined miR-126-3p expression by real-time PCR and found miR-126-3p was repressed by folic acid treatment. Epigenetic modulation of the miR-126-3p has been recently demonstrated [9,37].

Folate is required for one-carbon metabolism involving the transfer and utilization of one-carbon units in essential pathways. One-carbon metabolism is essential for de novo purine and thymidylate synthesis and for remethylation of homocysteine to methionine, which can then be adenosinylated to form the universal methyl donor SAM. One-carbon metabolism was also related to AD-like hallmarks (increased Aβ production) [21,38]. Previous studies have indicated that in neural cells or AD mice, folic acid supplement leads to higher availability of SAM and activities of DNA methyltransferases (DNMTs) [24,39], which are required for cellular methylation reactions. Therefore, altered miRNAs expression in present study may be related to the changing of methyl donor pool, along with increasing DNA methylation by folic acid administration. 

However, miR-339-5p expression showed a reverse trend compared to miR-126-3p. In a previous study we have revealed folic acid deficiency down-regulated miR-339-5p, which targets BACE1 [19]. In the current study, we demonstrated folic acid supplementation could up-regulate miR-339-5p and down-regulate BACE1 expression, which can further confirm the relationship between folic acid, miR-339-5p and BACE1. Since folic acid showed different effect on miRNAs which target different secretase, the underlying regulation mechanism of folic acid on miRNAs needs further investigation.

## 5. Conclusions

Our findings suggested that folic acid reduced Aβ42 content in APP/PS1 mice brains. Furthermore, β-secretase decreased significantly, and different subtypes or components of α-secretase (particularly ADAM9 and ADAM10) significantly increased. miRNAs which target ADAM9 and BACE1 may involve in the regulation effect of folic acid on those certain secretases.
